# Temporal trends in chronic complications of diabetes by sex in community-based people with type 2 diabetes: the Fremantle Diabetes Study

**DOI:** 10.1186/s12933-023-01980-8

**Published:** 2023-09-16

**Authors:** Wendy A. Davis, Timothy M. E. Davis

**Affiliations:** Medical School, University of Western Australia, Fremantle Hospital, P. O. Box 480, Fremantle, WA 6959 Australia

**Keywords:** Type 2 diabetes, Cardiovascular disease, Sex, Mortality, Temporal trends, Community-based, Longitudinal study

## Abstract

**Background:**

Whether recent reductions in cardiovascular disease (CVD) events and mortality in type 2 diabetes apply equally to both sexes is largely unknown. The aim of this study was to characterize temporal changes in CVD events and related outcomes in community-based male and female Australian adults with type 2 diabetes or without known diabetes.

**Methods:**

Participants from the longitudinal observational Fremantle Diabetes Study Phases I (FDS1; n = 1291 recruited 1993–1996) and II (FDS2; n = 1509 recruited 2008–2011) and four age-, sex- and postcode-matched individuals without diabetes (FDS1 n = 5159; FDS2 n = 6036) were followed for first myocardial infarction, stroke, heart failure hospitalization, lower extremity amputation, CVD death and all-cause mortality. Five-year incidence rates (IRs) for males versus females in FDS1 and FDS2 were calculated, and IR ratios (IRRs) derived.

**Results:**

The FD1 and FDS2 participants were of mean age 64.0 and 65.4 years, respectively, and 48.7% and 51.8% were males. For type 2 diabetes, IRRs for all endpoints were 11–62% lower in FDS2 than FDS1 for both sexes. For participants without diabetes, IRRs were 8–56% lower in FDS2 versus FDS1 apart from stroke in females (non-significantly 41% higher). IRRs for males versus females across FDS phases were not significantly different for participants with type 2 diabetes or those without diabetes (*P*-values for male * FDS2 interaction ≥ 0.0.083 adjusted for age). For risk factors in participants with type 2 diabetes, greater improvements between FDS1 and FDS2 in smoking rates in males were offset by a greater reduction in systolic blood pressure in females.

**Conclusions:**

The incidence of chronic complications in Australians with type 2 diabetes and without diabetes has fallen similarly in both sexes over recent decades, consistent with comparably improved overall CVD risk factor management.

**Supplementary Information:**

The online version contains supplementary material available at 10.1186/s12933-023-01980-8.

## Background

Data from a number of high-income countries have shown that there has been a decline in the incidence of the major chronic complications of diabetes over the last few decades [1–4]. This includes myocardial infarction (MI), stroke and lower extremity amputation (LEA), in addition to death due to cardiovascular disease (CVD) and all-cause mortality [5], and is thought to reflect concomitantly improved CVD risk factor management [6–8]. Although US studies suggest that trends in CVD risk factor control and events may have plateaued or perhaps even partially reversed in some groups of patients in recent years [9, 10], Australian data show that people with type 2 diabetes have increasingly fewer complications [11, 12].

Whether the recent encouraging trends in CVD events and mortality in type 2 diabetes applies equally to both sexes is largely unknown. Short-term Australian hospitalisation data from 2010 to 2019 suggest that women have had relatively better outcomes over time than men for MI, stroke, LEA and heart failure (HF) [12], but the temporal trends in these endpoints (other than LEA which was not included) were similar in males and females in a study from Hong Kong conducted over the same time period [13]. Neither of these studies included a parallel matched group of people without diabetes to assess whether sex-specific changes reflected diabetes-specific trends or simply those in the population as a whole.

In light of these considerations, we have used baseline and longitudinal outcome data collected between 1993 and 2016 inclusive from the community-based Fremantle Diabetes Study Phases I (FDS1) and II (FDS2) to determine whether: (i) the reductions in 5-year incidence rates (IRs) of major chronic complications and mortality in Australians with type 2 diabetes have differed by sex, and (ii) there have been parallel sex-specific changes in morbidity and mortality in matched people without known diabetes from the same geographical area.

## Methods

### Participants and approvals

The FDS1 is an observational, longitudinal study of clinician-diagnosed (except gestational) diabetes conducted in a postcode-defined geographic area surrounding the port city of Fremantle in the state of Western Australia (WA) [14, 15]. The recruitment period for FDS1 was between 1993 and 1996, with follow-up of diabetes complications and mortality data in the present sub-study to end-2013. The FDS2 utilized the same design as FDS1 [15], with recruitment between 2008 and 2011 and present follow-up to end-2016. Participants in FDS1 and FDS2 were identified from hospital and primary care patient lists, widespread local advertising, pharmacies, optometrists, health care professional networks, and, for FDS2, third-party mail-outs to registrants of the National Diabetes Services Scheme and the National Diabetes Register [15]. Details of recruitment, sample characteristics, and eligible but non-recruited people have been published [14, 15]. The FDS1 protocol was approved by the Fremantle Hospital Human Rights Committee, and the FDS2 protocol by the Human Research Ethics Committee of the Southern Metropolitan Area Health Service (reference 07/397). All participants gave written informed consent.

In FDS1, 2258 people with diabetes were identified from a population of approximately 120,000, and 1426 (63%) recruited of whom 1296 (91%) had clinician confirmed type 2 diabetes. In FDS2, 4639 people with diabetes were identified from a population of approximately 157,000, and 1668 (36%) recruited of whom 1509 (90%) had type 2 diabetes. Socio-economic data from the catchment area during FDS2 recruitment showed an average Index of Relative Socio-economic Advantage and Disadvantage [16] of 1033 with a range by postcode of 977–1113, figures comparable to the Australian national mean ± SD of 1000 ± 100. Four age-, sex- and postcode-matched residents without any prior record of diabetes on any WA administrative health database (see below) were randomly selected from the catchment area for each FDS1 and FDS2 participant at the time of their enrolment using the WA Electoral Roll and, for FDS2, the WA Registry for Births, Deaths and Marriages. After exclusion of FDS1 participants who were unable to be matched, 1291 with type 2 diabetes (99.6%) were matched with 5159 residents without diabetes and, in FDS2, all 1509 FDS2 participants with type 2 diabetes were matched with 6036 residents without diabetes. If the matched residents without diabetes developed diabetes during follow-up, they were censored at the time this was first recorded.

### Baseline and annual assessments

In both study Phases, assessment at entry and at each annual (FDS1) or biennial (FDS2) face-to-face review included a comprehensive questionnaire, physical examination and fasting biochemical tests performed in a single nationally accredited laboratory [14]. In FDS2, comprehensive postal questionnaires were sent to participants in the years between face-to-face assessments. Complications were identified using standard definitions [17]. Albuminuria was assessed by early morning spot urine albumin:creatinine ratio (ACR) measurement and renal impairment from the estimated glomerular filtration rate (eGFR) [18]. Peripheral sensory neuropathy (PSN) was defined using the clinical portion of the Michigan Neuropathy Screening Instrument [19]. Retinopathy was defined as one microaneurysm in either eye or worse and/or evidence of previous laser treatment on direct/indirect ophthalmoscopy (FDS1) or fundus photography (FDS2), and/or ophthalmologist assessment. Participants were classified as having prevalent coronary heart disease (CHD) if there was a history of MI, angina, coronary artery bypass grafting, or angioplasty, and as having prevalent cerebrovascular disease if there was a history of stroke and/or transient ischemic attack. Peripheral arterial disease (PAD) was defined as an ankle brachial index ≤ 0.90 or the presence of a diabetes-related lower extremity amputation (LEA).

### Ascertainment of outcomes

The outcomes of interest were first fatal or non-fatal MI, first fatal or non-fatal stroke, first hospital admission for/with heart failure (HF), first LEA, CVD mortality (death from cardiac or cerebrovascular causes or sudden death) and all-cause mortality. The Hospital Morbidity Data Collection (HMDC) contains validated information regarding all public/private hospitalizations in WA since 1970 and the Death Register contains information on all deaths in WA [20]. Both FDS phases have been linked to these databases through the WA Data Linkage System (WADLS), as approved by the WA Department of Health Human Research Ethics Committee, to provide validated data on incident events to end-2013 for FDS1 and end-2016 for FDS2. Relevant International Classification of Disease (ICD)-9-CM and ICD-10-AM codes were used to identify outcomes in the HMDC, as detailed previously [11]. Causes of death on the death certificate or coroner’s report were reviewed independently by two study physicians and classified under the system used in the UK Prospective Diabetes Study [21], as also described previously [11].

The HMDC was used to supplement data obtained through FDS assessments relating to prevalent/prior disease during the 5 years prior to study entry as well as providing the same information for the matched residents without diabetes. These data were used to calculate the Charlson Comorbidity Index (CCI) [22] which includes a history of MI, HF, PAD, cerebrovascular disease, chronic pulmonary disease, rheumatic disease, peptic ulcer disease, hemiparesis or paraparesis, renal disease, liver disease, and cancer. For the purposes of the present study, we excluded those conditions coded as diabetes-specific chronic complications (ICD-9-CM 250 and ICD-10-AM E10-14 codes) in FDS participants.

### Statistical analysis

The computer packages IBM SPSS Statistics 28 (IBM Corporation, Armonk, NY, USA) and StataSE 15 (College Station, TX: StataCorp LP) were used for statistical analysis. Data are presented as proportions, mean ± SD, geometric mean (SD range), or, in the case of variables which did not conform to a normal or log-normal distribution, median and inter-quartile range [IQR]. Two-sample comparisons were by Fisher’s exact test for proportions, Student’s *t*-test for normally distributed variables, and Mann–Whitney *U*-test for other variables. More than two independent samples were compared with the Fisher-Freeman-Halton exact test for proportions, ANOVA for normally or log_e_-normally distributed variables, or the Kruskal–Wallis test otherwise.

Five-year incidence rates (IRs) for each outcome were derived for each of the eight groups defined by sex, type 2 diabetes status and FDS Phase. IR ratios (IRRs) and IR differences (IRDs) were then calculated for (i) males with type 2 diabetes in FDS2 versus FDS1, (ii) females with type 2 diabetes in FDS2 versus FDS1, (iii) males without diabetes in FDS2 versus FDS1, (iv) females without diabetes in FDS2 versus FDS1, (v) males versus females in FDS1 participants with type 2 diabetes, (vi) males versus females in FDS2 participants with type 2 diabetes, (vii) males versus females in FDS1 participants without diabetes, (viii) males versus females in FDS2 participants without diabetes. IRs were compared by unadjusted Poisson regression with log_e_(time) as the exposure variable. Poisson regression was also used to determine whether there was any interaction between IRs by sex and FDS phase, separately for type 2 diabetes and no diabetes, unadjusted and adjusted for age. The FDS1 and FDS2 participants with type 2 diabetes and without diabetes were pooled. IRRs for all outcomes were ascertained in multivariable Poisson regression models for sex, diabetes status, and FDS phase, including two- and three-way interactions, with adjustment for age. In the type 2 diabetes cohorts only, logistic and linear regression with sex, FDS phase and their interaction entered as independent variables were used to determine whether changes in dichotomous and continuous risk factors, respectively, across time (FDS phase) differed by sex.

## Results

### Participant characteristics

The total sample of 13,995 FDS1 and FDS2 participants combined with the two matched cohorts without diabetes had a mean ± SD age of 64.8 ± 11.5 years and 50.4% were males. The matched cohorts in FDS2 were slightly older and more likely to be male than those in FDS1 (mean age 65.4 versus 64.0 years, 51.8% versus 48.7%; *P* ≤ 0.005).

### Outcomes by FDS phase, type 2 diabetes status and sex

**Fig. 1:**
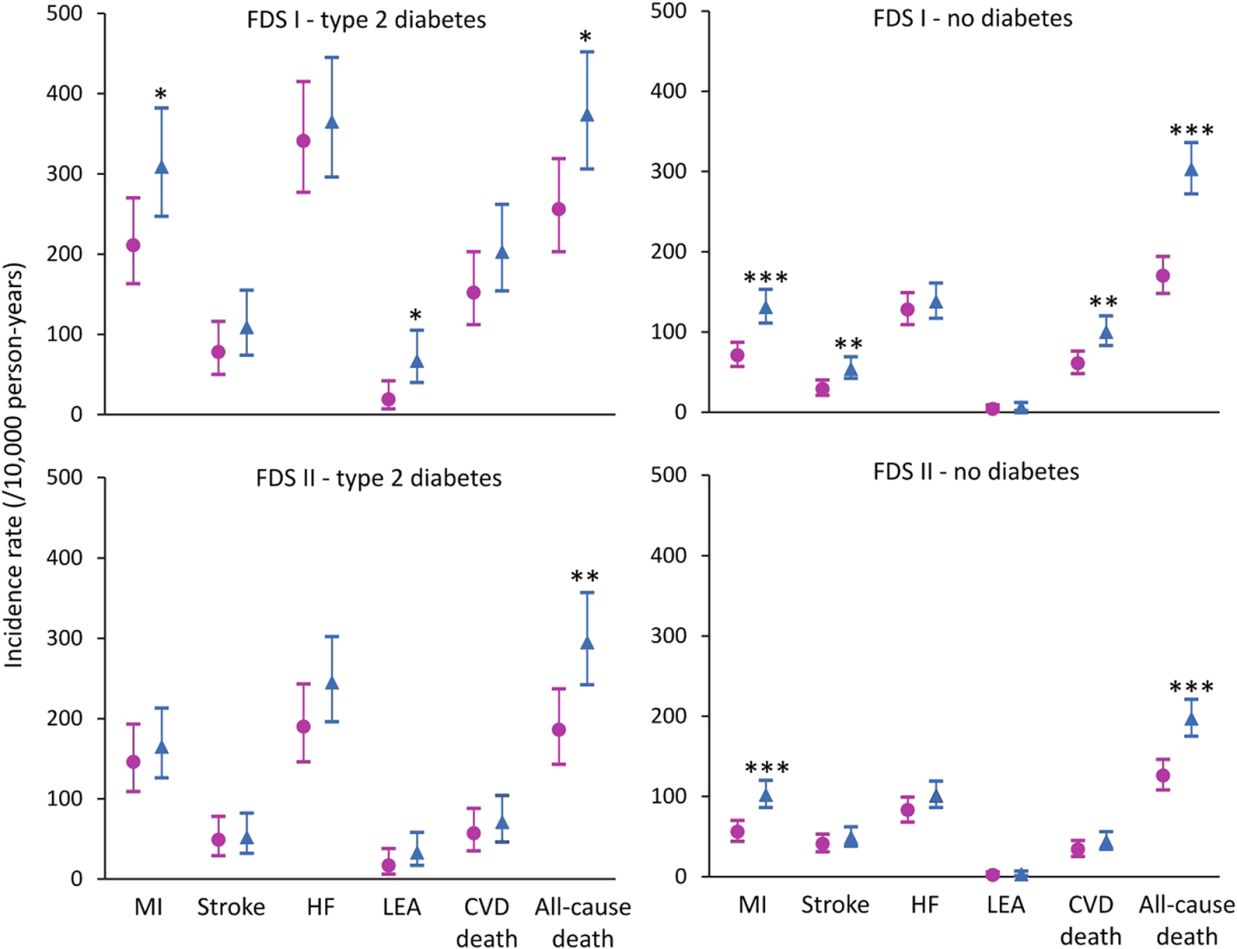
Incidence rates and 95% confidence intervals (vertical bars) for key outcomes in FDS1 and FDS2 by sex (pink circle females, blue diamond males) for participants with type 2 diabetes (left hand panels) and without recorded diabetes at baseline (right hand panel). *P < 0.05; ** *P* < 0.01, *** *P* < 0.001 vs females

**Fig. 2:**
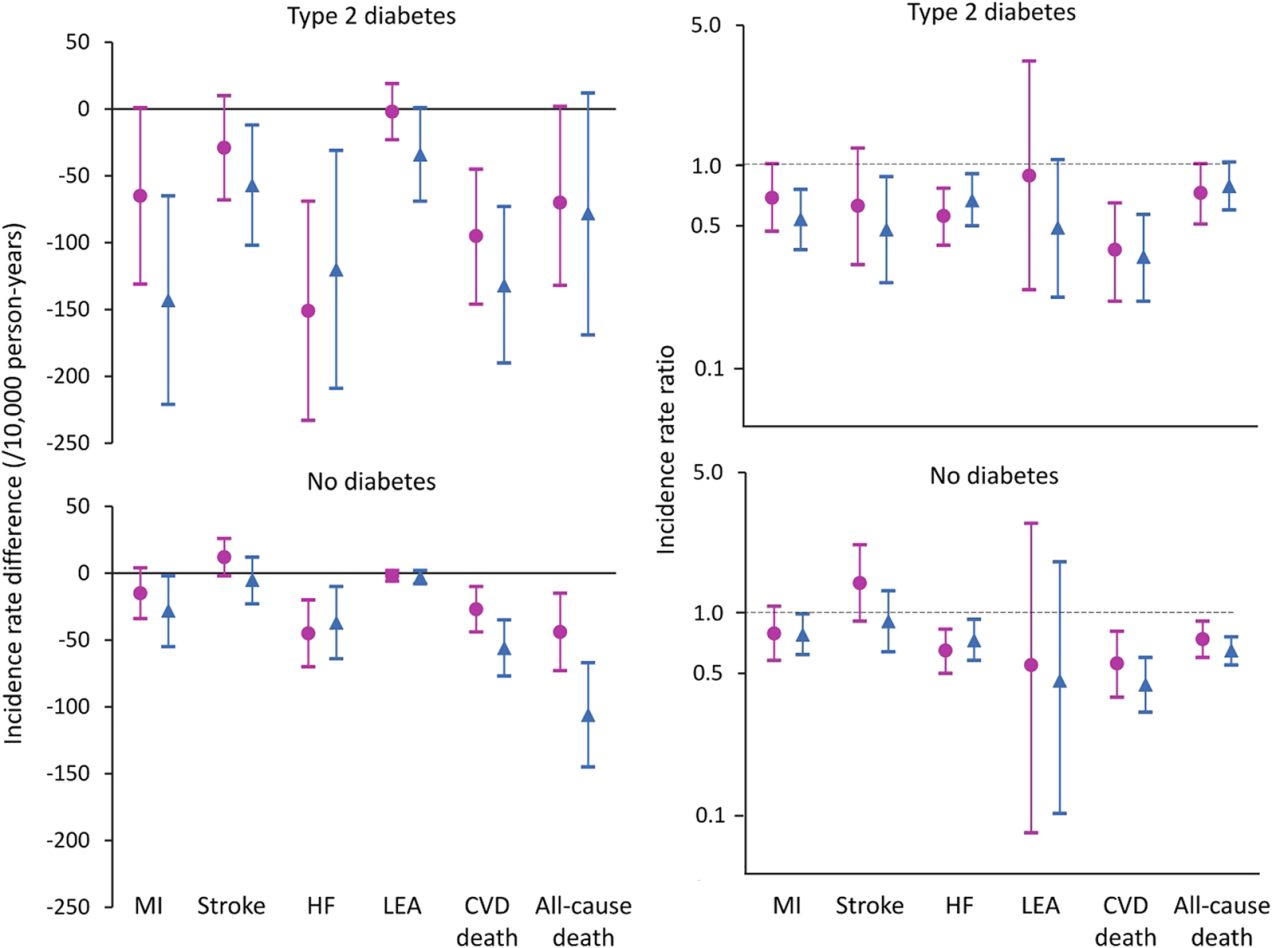
Incidence rate differences between FDS1 and FDS2 (left hand panels) and FDS2:FDS1 incidence rate ratios (right hand panels, logarithmic scale) with 95% confidence intervals (vertical bars) for key outcomes by sex (pink circle females, blue diamond males) for participants with type 2 diabetes (upper panels) and without recorded diabetes at baseline (lower panels)

In FDS1, the 5-year IRs for males with type 2 diabetes were statistically significantly higher than females for MI, LEA, and all-cause mortality while, in FDS2, only all-cause death was significantly higher in males (see Fig. 1 and Additional file 1: Table S1). In the case of the matched individuals without diabetes, there was a significant male preponderance for MI, stroke, CVD death and all-cause mortality during follow-up in FDS1, and for MI and all-cause death in FDS2 (see Fig. 1 and Additional file 1: Table S1). For participants with type 2 diabetes, all IRDs for FDS2 versus FDS1 were negative and all IRRs were less than unity for both sexes (see Fig. 2 and Additional file 1: Table S1). This was also the case for participants without diabetes except for stroke in females but the 95% CIs for the respective IRD and IRR in this case spanned zero and unity, respectively (see Fig. 2 and Additional file 1: Table S1).

**Fig. 3:**
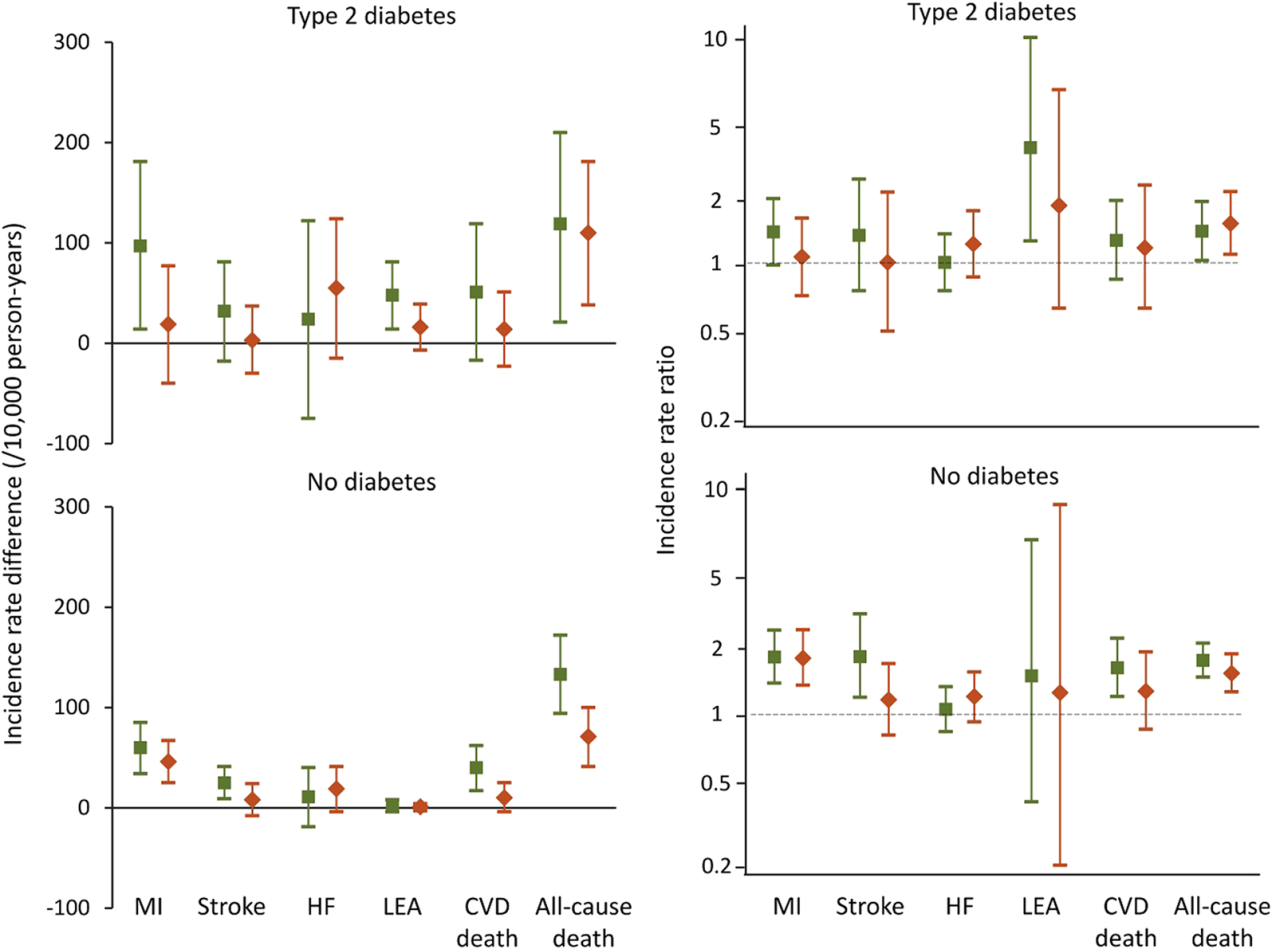
Incidence rate differences between males and females (left hand panels) and male:female incidence rate ratios (right hand panels, logarithmic scale) with 95% confidence intervals (vertical bars) for key outcomes in FDS1 (green sqaure) and FDS2 (orange diamond) for participants with type 2 diabetes (upper panels) and without recorded diabetes at baseline (lower panels)

There was a reduction in the incidence of all outcomes in FDS2 versus FDS1 regardless of sex and diabetes status. However, both IRDs between males and females and male:female IRRs in FDS1 versus FDS2 were not significantly different for participants with type 2 diabetes and those without (*P*-values for interaction male*FDS2 ≥ 0.109 unadjusted and ≥ 0.0.083 adjusted for age; see Fig. 3 and Additional file 1: Table S2).

**Fig. 4:**
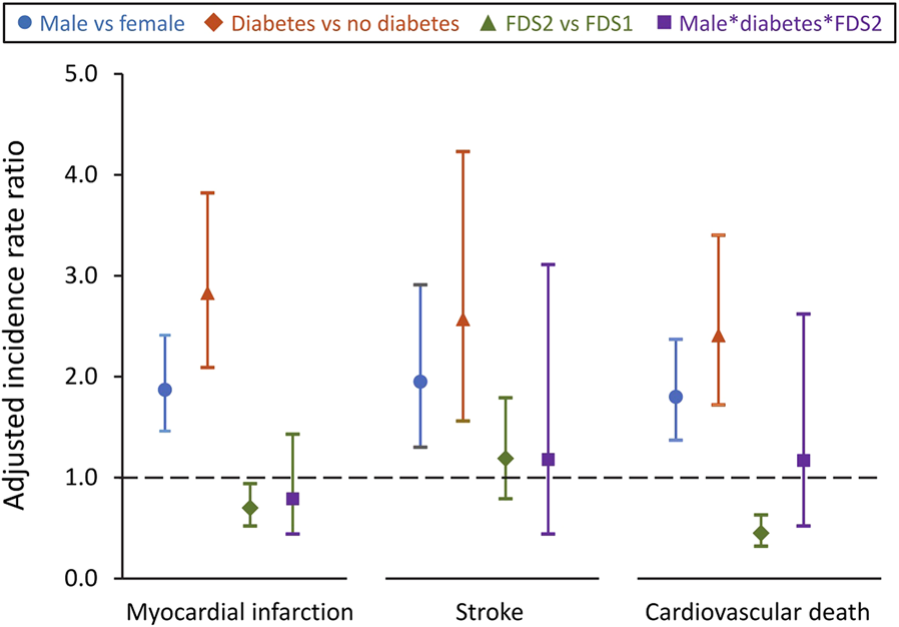
Five-year incidence rate ratios for myocardial infarction, stroke and cardiovascular mortality in pooled FDS1 and FDS2 participants with type 2 diabetes and without diabetes analysed separately by main effects (sex, diabetes status, FDS phase) and their 3-way interaction, adjusted for age, the other main effects, two-way interactions, and, for the main effects, three-way interaction (see Table 1)

The 5-year IRRs for all outcomes in the pooled sample of all participants with inclusion of main effects (sex, diabetes status and FDS phase) and their interactions terms with adjustment for age is shown in Table 1. Overall, males had nearly double the incidence of MI and stroke than females, but the IRRs for heart failure and LEA were not statistically different by sex. Individuals with type 2 diabetes in FDS2 had a significantly lower IRR for stroke than their counterparts in FDS1, but this was also independent of sex. Participants in FDS2 had a lower incidence of MI, HF, CVD death and all-cause mortality than in FDS1 (reductions of 30%, 46%, 55% and 38%, respectively, after adjustment for age) regardless of diabetes status and sex. Age had no influence on the IRR for LEAs. The adjusted IRRs for the three major adverse cardiovascular events MI, stroke and CVD death are shown in Fig. 4. The confidence intervals for the interaction term male * diabetes * FDS2 crossed unity for each outcome.

**Table 1:** Five-year incidence rate ratios (IRR) for outcomes in pooled FDS1 and FDS2 participants with type 2 diabetes and without diabetes including main effects (sex, diabetes status, FDS phase) and their two- and three-way interactions adjusted for age

	MI		Stroke		HF		LEA		CVD death		All-cause death	
	IRR (95% CI)	*P*-value	IRR (95% CI)	*P*-value	IRR (95% CI)	*P*-value	IRR (95% CI)	*P*-value	IRR (95% CI)	*P*-value	IRR (95% CI)	*P*-value
Age (1 year increase)	1.07 (1.06, 1.08)	< 0.001	1.09 (1.08, 1.10)	< 0.001	1.10 (1.09, 1.11)	< 0.001	1.01 (0.99, 1.04)	0.292	1.11 (1.10, 1.12)	< 0.001	1.10 (1.09, 1.10)	< 0.001
Male (vs female)	1.87 (1.46, 2.41)	< 0.001	1.95 (1.30, 2.91)	0.001	1.15 (0.94, 1.42)	0.167	1.06 (0.31, 3.65)	0.928	1.80 (1.37, 2.37)	< 0.001	1.90 (1.63, 2.21)	< 0.001
Diabetes (vs no diabetes)	2.83 (2.09, 3.82)	< 0.001	2.57 (1.56, 4.23)	< 0.001	2.47 (1.97, 3.10)	< 0.001	4.79 (1.47, 15.6)	0.009	2.41 (1.72, 3.40)	< 0.001	1.45 (1.15, 1.83)	0.001
FDS2 (vs FDS1)	0.70 (0.52, 0.94)	0.016	1.19 (0.79, 1.79)	0.394	0.54 (0.43, 0.68)	< 0.001	0.54 (0.13, 2.24)	0.392	0.45 (0.32, 0.63)	< 0.001	0.62 (0.52, 0.75)	< 0.001
Male * diabetes	0.80 (0.54, 1.17)	0.251	0.76 (0.40, 1.46)	0.418	1.00 (0.73, 1.39)	0.977	2.84 (0.61, 13.3)	0.186	0.82 (0.52, 1.29)	0.391	0.83 (0.62, 1.12)	0.226
Male * FDS2	0.97 (0.67, 1.39)	0.863	0.63 (0.37, 1.06)	0.083	1.10 (0.81, 1.50)	0.544	1.17 (0.17, 8.18)	0.873	0.77 (0.48, 1.21)	0.251	0.85 (0.68, 1.07)	0.163
Diabetes * FDS2	0.91 (0.58, 1.43)	0.673	0.46 (0.22, 0.96)	0.037	0.91 (0.63, 1.31)	0.603	1.67 (0.27, 10.3)	0.581	0.70 (0.38, 1.30)	0.260	1.02 (0.72, 1.43)	0.928
Male * diabetes * FDS2	0.79 (0.44, 1.43)	0.433	1.18 (0.44, 3.11)	0.744	1.05 (0.64, 1.72)	0.860	0.53 (0.05, 5.60)	0.596	1.17 (0.52, 2.62)	0.710	1.22 (0.79, 1.89)	0.361

The baseline characteristics of the participants with type 2 diabetes in FDS1 and FDS2 categorised by sex are summarised in Table 2. In relation to modifiable CVD risk factors, although current smoking rates were significantly higher in males than females irrespective of FDS phase (*P* = 0.001), never smoking rates were significantly higher for males in FDS2 than FDS1 (male*FDS2 interaction, *P* < 0.001). Glycaemic control was significantly better in FDS2 relative to FDS1 but there was no sex difference (male * FDS2 interaction for HbA_1c_, *P* = 0.395). There was an increase in obesity in FDS2 but to a similar extent by sex (male * FDS2 interaction for BMI, *P* = 0.090). Systolic blood pressure was lower in FDS2 compared with FDS1, especially in females (male * FDS2 interaction, *P* = 0.003). Males were less likely than females to be on antihypertensive medications in FDS1, but were as likely to be treated with them in FDS2 (male * FDS2 interaction, *P* < 0.001). Reductions in serum total cholesterol in FDS2 were no greater in males (male * FDS2 interaction, *P* = 0.957), but males had lower levels than females in both phases (*P* < 0.001). There was no male * FDS2 interaction (*P* = 0.127) in the use of lipid lowering medications.

**Table 2:** Baseline characteristics of participants with type 2 diabetes in FDS1 and FDS2 by sex

	FDS1		FDS2		*P*-value
	Females	Males	Females	Males	
Number (%)	666 (23.7)	630 (22.5)	727 (25.9)	782 (27.9)	
Age at entry (years)	64.2 ± 11.8	63.9 ± 10.7	65.3 ± 12.2	65.5 ± 11.2^†^	0.012
Age at diagnosis (years)	58.0 ± 11.9	57.7 ± 11.5	55.7 ± 13.0^**,†^	55.5 ± 11.7^***,††^	< 0.001
Duration of diabetes (years)	4.0 [1.0–9.0]	4.0 [1.0–9.3]	8.0 [3.0–15.5]^***^	9.0 [2.1–15.4] ^***,†††^	< 0.001
Ethnic background (%)			^†††^	^†††^	< 0.001
Anglo-Celt	61.1	61.7	53.5	51.7	
Southern European	18.6	16.8	11.6	14.1	
Other European	7.7	9.4	6.7	7.9	
Asian	3.5	3.3	3.9	4.7	
I ndigenous Australian	1.8	1.1	9.6	4.7	
Mixed/other	7.4	7.6	14.7	16.9	
Not fluent in English (%)	16.8	13.7	10.9^**^	10.7^**^	0.002
Education beyond primary level (%)	70.2	78.0	86.4^†††^	87.1^†††^	< 0.001
Currently married/de facto (%)	57.8	73.9^***^	52.5^†††^	72.1^***,‡‡‡^	< 0.001
Alcohol (standard drinks/day)	0 [0–0.1]	0.3 [0–1.5]^***^	0 [0–0.3]^***^	0.3 [0–1.5]^***,†††^	< 0.001
Smoking status (%)		^***^	^†††^	^***,†††,‡‡‡^	< 0.001
Never	64.2	24.2	60.0	32.0	
Ex-	23.9	57.3	30.3	56.4	
Current	11.9	18.5	9.7	11.6	
Diabetes treatment (%)			^***^	^***,†††,‡‡^	< 0.001
Diet	31.5	32.4	28.8	20.7	
Oral agents or non-insulin injectables	56.0	55.4	50.1	56.4	
Insulin ± oral agents or non-insulin injectables	12.5	12.1	21.1	22.9	
Fasting serum glucose (mmol/L)	8.0 [6.4–10.4]	7.9 [6.5–10.2]	7.1 [6.1–8.8]^***^	7.2 [6.2–9.0] ^***,†††^	< 0.001
HbA_1c_ (%)	7.2 [6.2–8.6]	7.2 [6.2–8.5]	6.8 [6.2–7.7]^***^	6.9 [6.2–7.8] ^***,^^†^	< 0.001
HbA_1c_ (mmol/mol)	55 [44–71]	55 [44–69]	51 [44–61]	52 [44–62]	< 0.001
BMI (kg/m^2^)	30.3 ± 6.1	28.8 ± 4.6^***^	31.7 ± 6.7^***,†††^	30.9 ± 5.6^†††^	< 0.001
Systolic blood pressure (mm Hg)	151 ± 24	151 ± 23	143 ± 24^***,†††^	148 ± 21^‡‡‡^	< 0.001
Diastolic blood pressure (mm Hg)	79 ± 11	82 ± 11^***^	77 ± 13^†††^	83 ± 12^***,‡‡‡^	< 0.001
Antihypertensive medication (%)	56.0	45.6^**^	71.9^***^	74.4^***,†††^	< 0.001
Heart rate (bpm)	72 ± 12	68 ± 13^***^	71 ± 12^†††^	68 ± 12^***,‡‡‡^	< 0.001
Serum total cholesterol (mmol/L)	5.7 ± 1.1	5.2 ± 1.0^***^	4.6 ± 1.1^***,†††^	4.1 ± 1.0^***,†††,‡‡‡^	< 0.001
Serum HDL-cholesterol (mmol/L)	1.14 ± 0.34	0.97 ± 0.30^***^	1.34 ± 0.35^***,†††^	1.14 ± 0.30^†††,‡‡‡^	< 0.001
Total cholesterol:HDL-cholesterol ratio	5.1 (3.6–7.1)	5.5 (4.0–7.6)^***^	3.4 (2.5–4.7)^***,†††^	3.6 (2.7–4.9)^***,†††,‡‡^	< 0.001
Serum triglycerides (mmol/L)	2.2 (1.3–3.7)	2.2 (1.2–4.0)	1.5 (0.9–2.5)^***,†††^	1.5 (0.9–2.6)^***,†††^	< 0.001
Lipid-modifying medication (%)	11.1	9.9	66.0^***,†††^	70.2^***,†††^	< 0.001
Aspirin use (%)	18.9	25.2^*^	34.4^***,††^	39.8^***,†††^	< 0.001
Urinary albumin:creatinine (mg/mmol)	5.4 (1.7–17.0)	5.0 (1.3–18.6)	3.2 (0.9–11.6)^***,†††^	3.3 (0.8–13.7)^***,†††^	< 0.001
Estimated glomerular filtration rate (%)				^†^	0.003
≥ 90 mL/min/1.73m^2^	32.3	32.1	40.2	37.8	
60–89 mL/min/1.73m^2^	48.1	51.6	43.8	45.3	
45–59 mL/min/1.73m^2^	12.5	11.3	8.9	8.7	
30–44 mL/min/1.73m^2^	5.2	3.5	5.0	4.9	
< 30 mL/min/1.73m^2^	2.0	1.4	2.2	3.3	
Atrial fibrillation (%)	3.2	6.6^*^	3.5	5.6	0.008
Ischaemic heart disease (%)	26.1	33.2^*^	24.8^††^	32.6^*,^^‡‡^	< 0.001
Cerebrovascular disease (%)	8.9	11.1	9.9	12.4	0.15
Peripheral arterial disease (%)	28.9	29.8	26.8^†††^	18.7^***,‡‡^	< 0.001
Peripheral sensory neuropathy (%)	27.0	34.7^*^	56.0^***,†††^	60.2^***,†††^	< 0.001
Charlson Comorbidity Index^a^ (%)			^*^		< 0.001
0	74.0	68.9	78.3	72.3	
1–2	20.4	23.8	14.3	19.2	
≥ 3	5.6	7.3	7.4	8.6	

^*^P < 0.05, ^**^P < 0.01, ^***^P < 0.001 vs FDS1 females

^†^*P* < 0.05, ^††^*P* < 0.01, ^†††^*P* < 0.001 vs FDS1 males

^‡^*P* < 0.05, ^‡‡^*P* < 0.01, ^‡‡‡^*P* < 0.001 vs FDS2 females, Bonferroni-corrected for multiple comparisons

^a^In the last 5 years, excluding diabetes and its complications

## Discussion

The present data show that there were no clear and consistent differences between community-based males and females with type 2 diabetes in the reductions in 5-year incidence rates of major complications and mortality observed over the 15 years between FDS1 and FDS2. This finding also applied to a larger matched sample of individuals without diabetes living in the study catchment area and followed for these outcomes over the same time period. The proportionate reductions in CVD and related events between study phases in the participants with type 2 diabetes and in those without diabetes were similar (11–62% and 8–56%, respectively, apart from stroke in females without diabetes which was 41% higher in FDS2). There were significant between-sex differences between FDS1 and FDS2 in baseline modifiable CVD risk factors in the participants with type 2 diabetes, but more favourable improvements in never smoking rates in males versus females during follow-up were offset by a greater reduction in systolic blood pressure in females versus males.

### Comparison with previously published sex-specific chronic complication data in type 2 diabetes

These observations complement and extend the limited data published to date that have examined potential sex differences in temporal changes in major diabetes complications [12, 13]. In a study from Hong Kong, the rate of decline between 2010 and 2019 in overall CVD, coronary heart disease, stroke and HF was similar in males and females [13], as observed in the present study over a longer period. In a large administrative database study from Australia also conducted between 2010 and 2019 [12], the incidence of stroke and LEA increased over time and this was greater in males than females, and there was a greater relative decrease in the incidence of MI and HF in females than males. These findings differ from those of the present study and may relate to the shorter duration and acknowledged limitations of the latter study [12] including potential miscoding of type of diabetes, absence of private hospitalisation data in the ascertainment of events, and accuracy of coding of endpoints. In the present study, type of diabetes was relatively strictly defined [15], all private hospitalisations were captured, and the WADLS has been well validated for the cardiovascular and other outcomes of interest [23–27].

### Sex-specific changes in mortality in type 2 diabetes

There have also been discrepancies between studies in relation to sex-specific differences in mortality [28]. In the Hong Kong study [13], all-cause death increased in parallel in both sexes. By contrast, all-cause death was reduced from 2002 to 2014 by 29% in males and 17% in females with type 2 diabetes in Australia [29]. In large administrative database studies of diabetes of unspecified type, all-cause mortality decreased by 32% in males and 31% in females from 2001 to 2018 in a UK study [30] and there were 12% and 14% reductions in males and females, respectively, from 2005 to 2014 in a Taiwanese study [31]. Data from these latter three studies [29–31] are largely consistent with those of the present study showing a decline in all-cause death without a significant sex difference.

### Temporal sex-specific changes in outcomes in people without diabetes

Consistent with general population data from Western Australia collected between 1995 and 2010 [32], rates of CVD outcomes declined in both sexes in our participants who did not have diabetes, albeit with higher IRs in males. We did not have detailed data relating to risk factors and their management in this group corresponding to those available for the FDS participants with type 2 diabetes. However, there is evidence from Australian population-based data sources that the uptake of cardiovascular risk-reducing medications including statins and antihypertensives has been increasing over recent decades [33, 34], in accord with our outcome data. In the case of our participants with type 2 diabetes, there was no evidence that use of these medications was significantly lower in females compared with males in either FDS1 or FDS2, in contrast to lower rates of prescription in females in the general Australian population [35]. This may represent increasing recognition of the loss of the protective effect of female sex on CVD events in diabetes [36].

### Limitations and strengths of the present study

Although the age, sex and proportions by diabetes type of participants and non-participants in both FDS1 and FDS2 were similar [15], healthier residents with diabetes may have participated. Although the WADLS is regularly validated [20], and the coding of key outcomes in the present study appears robust [23–27], there may have been misclassification. Some participants in the matched cohort without diabetes might have had diabetes at baseline which had not yet been coded in databases linked through the WADLS. Both FDS phases were conducted before the widespread availability of the glucagon-like peptide 1 receptor agonists and sodium-glucose co-transporter-2 inhibitors in Australia. Although there may be sex-specific differences in uptake and tolerability with these agents, there is no evidence of a difference in cardiovascular endpoints [37]. Since the present analyses involved time to first event, we did not consider post-event outcomes in which there may be sex-specific differences [38, 39]. The strengths of the present study are the large samples of participants followed for a long period, its well characterized participants with type 2 diabetes, and linkage to comprehensive health databases through the WADLS. The socioeconomic similarity between the FDS catchment area and the general Australian population suggest that the present findings reflect national trends, but broader application is questionable given between-country epidemiological differences.

## Conclusions

The 5-year incidence rates of CVD events and related outcomes in representative, community-based cohorts of Australians with type 2 diabetes and from the general population have declined similarly in both sexes since the 1990s. Improvements in CVD risk factor management are a likely key contributing factor without clear differences in overall management between males and females with type 2 diabetes. Surveillance of trends by sex should be an important consideration in the changing epidemiology of the chronic complications of diabetes [9, 10].

### Supplementary Information

**Additional file 1: Table S1.** Five-year incidence rates (IR; per 10,000 person-years), incidence rate ratios (IRR) and incidence rate differences (IRD; /10,000 person-years) for all-cause and cardiovascular disease mortality and chronic complications in FDS2 versus FDS1 type 2 diabetes participants and matched cohorts without diabetes by sex.** Table S2.** Five-year incidence rate ratios (IRR) and incidence rate differences (IRD; per 10,000 person-years) for all-cause and cardiovascular disease mortality and chronic complications by sex and phase in the type 2 diabetes and the no diabetes cohorts.

## Data Availability

Some outcome data supporting the findings of this study are available from the Western Australian Department of Health, but restrictions apply to the availability of these data, which were used under strict conditions of confidentiality for the current study, and so are not publicly available. Data are however available from the authors upon reasonable request and with permission of Western Australian Department of Health.
