# Supplementary material for: Temporal trends in chronic complications of diabetes by sex in community-based people with type 2 diabetes: the Fremantle Diabetes Study
Source: Cardiovasc Diabetol. 2023 Sep 16;22:253. doi: 10.1186/s12933-023-01980-8 (PMC10505315; doi:10.1186/s12933-023-01980-8)
Supplement: Supplementary file 1 — Additional file 1: Table S1. Five-year incidence rates (IR; per 10,000 person-years), incidence rate ratios (IRR) and incidence rate differences (IRD; /10,000 person-years) for all-cause and cardiovascular disease mortality and chronic complications in FDS2 versus FDS1 type 2 diabetes participants and matched cohorts without diabetes by sex. Table S2. Five-year incidence rate ratios (IRR) and incidence rate differences (IRD; per 10,000 person-years) for all-cause and cardiovascular disease mortality and chronic complications by sex and phase in the type 2 diabetes and the no diabetes cohorts. [file 12933_2023_1980_MOESM1_ESM.docx]

**Table S1.** Five-year incidence rates (IR; per 10,000 person-years), incidence rate ratios (IRR) and incidence rate differences (IRD; /10,000 person-years) for all-cause and cardiovascular disease mortality and chronic complications in FDS2 versus FDS1 type 2 diabetes participants and matched cohorts without diabetes by sex.

|  | FDS1 |  | FDS2 |  | FDS2:FDS1 | FDS2-FDS1 |
| --- | --- | --- | --- | --- | --- | --- |
|  | Number | IR (95% CI) | Number | IR (95% CI) | IRR (95% CI) | IRD (95% CI) |
| Males |  |  |  |  |  |  |
| Participants with diabetes | 629 |  | 782 |  |  |  |
| All-cause mortality | 107 | 374 (306-452) | 108 | 295 (242-357) | 0.79 (0.60-1.04) | -78 (-169 to 12) |
| Cardiovascular disease mortality | 58 | 203 (154-262) | 26 | 71 (46-104) | 0.35 (0.21-0.57) | -132 (-190 to -73) |
| Fatal or non-fatal myocardial infarction | 85 | 309 (247-382) | 59 | 165 (126-213) | 0.54 (0.38-0.76) | -143 (-221 to -65) |
| Fatal or non-fatal stroke | 31 | 109 (74-155) | 19 | 52 (32-82) | 0.48 (0.26-0.88) | -57 (-102 to -12) |
| Hospitalization for/with heart failure | 98 | 365 (296-445) | 86 | 245 (196-302) | 0.67 (0.50-0.91) | -120 (-209 to -31) |
| Hospitalization for lower extremity amputation | 19 | 67 (40-105) | 12 | 33 (17-58) | 0.49 (0.22-1.07) | -34 (-69 to 1) |
| Participants without diabetes | 2514 |  | 3125 |  |  |  |
| All-cause mortality | 354 | 303 (272-336) | 287 | 197 (175-221) | 0.65 (0.55-0.76) | -106 (-145 to -67) |
| Cardiovascular disease mortality | 117 | 100 (83-120) | 64 | 44 (34-56) | 0.44 (0.32-0.60) | -56 (-77 to -35) |
| Fatal or non-fatal myocardial infarction | 151 | 131 (111-153) | 147 | 102 (86-120) | 0.78 (0.62-0.99) | -28 (-55 to -2) |
| Fatal or non-fatal stroke | 63 | 54 (42-69) | 71 | 49 (38-62) | 0.91 (0.64-1.29) | -5 (-23 to 12) |
| Hospitalisation for/with heart failure | 158 | 138 (117-161) | 146 | 101 (86-119) | 0.73 (0.58-0.93) | -37 (-64 to -10) |
| Hospitalisation for lower extremity amputation | 7 | 6 (2-12) | 4 | 3 (1-7) | 0.46 (0.10-1.80) | -3 (-8 to 2) |
| Females |  |  |  |  |  |  |
| Participants with diabetes | 662 |  | 727 |  |  |  |
| All-cause mortality | 79 | 256 (203-319) | 65 | 186 (143-237) | 0.73 (0.51-1.02) | -70 (-143 to 2) |
| Cardiovascular disease mortality | 47 | 152 (112-203) | 20 | 57 (35-88) | 0.38 (0.21-0.65) | -95 (-146 to -45) |
| Fatal or non-fatal myocardial infarction | 64 | 211 (163-270) | 50 | 146 (109-193) | 0.69 (0.47-1.02) | -65 (-131 to 1) |
| Fatal or non-fatal stroke | 24 | 78 (50-116) | 17 | 49 (29-78) | 0.63 (0.32-1.22) | -29 (-68 to 10) |
| Hospitalization for/with heart failure | 99 | 341 (277-415) | 64 | 190 (146-243) | 0.56 (0.40-0.77) | -151 (-233 to -69) |
| Hospitalization for lower extremity amputation | 6 | 19 (7-42) | 6 | 17 (6-38) | 0.89 (0.24-3.32) | -2 (-23 to 19) |
| Participants without diabetes | 2645 |  | 2911 |  |  |  |
| All-cause mortality | 216 | 170 (148-194) | 176 | 126 (108-146) | 0.74 (0.60-0.91) | -44 (-73 to -15) |
| Cardiovascular disease mortality | 77 | 61 (48-76) | 47 | 34 (25-45) | 0.56 (0.38-0.81) | -27 (-44 to -10) |
| Fatal or non-fatal myocardial infarction | 90 | 71 (57-87) | 78 | 56 (44-70) | 0.79 (0.58-1.08) | -15 (-34 to 4) |
| Fatal or non-fatal stroke | 37 | 29 (21-40) | 57 | 41 (31-53) | 1.41 (0.91-2.19) | 12 (-2 to 26) |
| Hospitalisation for/with heart failure | 159 | 128 (109-149) | 114 | 83 (68-99) | 0.65 (0.50-0.83) | -45 (-70 to -20) |
| Hospitalisation for lower extremity amputation | 5 | 4 (1-9) | 3 | 2 (0-6) | 0.55 (0.08-2.80) | -2 (-6 to 2) |

**Table S2.** Five-year incidence rate ratios (IRR) and incidence rate differences (IRD; per 10,000 person-years) for all-cause and cardiovascular disease mortality and chronic complications by sex and phase in the type 2 diabetes and the no diabetes cohorts.

|  | Males : females | |  |  | Males - females | |
| --- | --- | --- | --- | --- | --- | --- |
|  | FDS1 | FDS2 |  |  | FDS1 | FDS2 |
| Outcome | IRR (95% CI) | IRR (95% CI) | *P*-value^1^ | *P*-value^2^ | IRD (95% CI) | IRD (95% CI) |
| Type 2 diabetes |  |  |  |  |  |  |
| All-cause mortality | 1.47 (1.09-1.99) | 1.59 (1.16-2.20) | 0.729 | 0.814 | 119 (29 to 210) | 110 (38 to 181) |
| Cardiovascular disease mortality | 1.34 (0.90-2.01) | 1.24 (0.67-2.35) | 0.845 | 0.756 | 51 (-17 to 119) | 14 (-23 to 51) |
| Fatal or non-fatal myocardial infarction | 1.46 (1.04-2.05) | 1.13 (0.76-1.68) | 0.327 | 0.279 | 97 (14 to 181) | 19 (-40 to 77) |
| Fatal or non-fatal stroke | 1.41 (0.80-2.50) | 1.07 (0.53-2.19) | 0.532 | 0.469 | 32 (-18 to 81) | 3 (-30 to 37) |
| Hospitalisation for/with heart failure | 1.07 (0.80-1.43) | 1.29 (0.92-1.81) | 0.375 | 0.442 | 24 (-75 to 122) | 55 (-15 to 124) |
| Hospitalisation for lower extremity amputation | 3.45 (1.33-10.6) | 1.91 (0.67-6.22) | 0.491 | 0.494 | 48 (14 to 81) | 16 (-7 to 39) |
| No diabetes |  |  |  |  |  |  |
| All-cause mortality | 1.78 (1.50-2.12) | 1.56 (1.29-1.90) | 0.304 | 0.160 | 133 (94 to 172) | 71 (41-100) |
| Cardiovascular disease mortality | 1.65 (1.23-2.23) | 1.30 (0.88-1.94) | 0.335 | 0.245 | 40 (17 to 62) | 10 (-4 to 25) |
| Fatal or non-fatal myocardial infarction | 1.84 (1.41-2.42) | 1.82 (1.38-2.43) | 0.981 | 0.844 | 60 (34 to 85) | 46 (25 to 67) |
| Fatal or non-fatal stroke | 1.85 (1.22-2.86) | 1.19 (0.83-1.72) | 0.109 | 0.083 | 25 (9 to 41) | 8 (-8 to 24) |
| Hospitalisation for/with heart failure | 1.08 (0.86-1.36) | 1.23 (0.95-1.58) | 0.420 | 0.567 | 11 (-19 to 40) | 19 (-4 to 41) |
| Hospitalisation for lower extremity amputation | 1.52 (0.42-6.09) | 1.28 (0.22-8.72) | 0.867 | 0.893 | 2 (-4 to 8) | 1 (-3 to 4) |

^1^*P*-value for interaction Male*FDS2; ^2^age-adjusted *P*-value for interaction Male*FDS2, derived from Poisson regression including main effects and interaction
